# Redox-Mediated Post-Translational Modifications of Proteolytic Enzymes and Their Role in Protease Functioning

**DOI:** 10.3390/biom10040650

**Published:** 2020-04-23

**Authors:** Anastasiia I. Petushkova, Andrey A. Zamyatnin

**Affiliations:** 1 Institute of Molecular Medicine, Sechenov First Moscow State Medical University, 119991 Moscow, Russia; asyapeti@gmail.com; 2 Belozersky Institute of Physico-Chemical Biology, Lomonosov Moscow State University, 119992 Moscow, Russia

**Keywords:** proteases, reactive oxygen species, regulated cell death, apoptosis, autophagy, caspases, homeostasis

## Abstract

Proteolytic enzymes play a crucial role in metabolic processes, providing the cell with amino acids through the hydrolysis of multiple endogenous and exogenous proteins. In addition to this function, proteases are involved in numerous protein cascades to maintain cellular and extracellular homeostasis. The redox regulation of proteolysis provides a flexible dose-dependent mechanism for proteolytic activity control. The excessive reactive oxygen species (ROS) and reactive nitrogen species (RNS) in living organisms indicate pathological conditions, so redox-sensitive proteases can swiftly induce pro-survival responses or regulated cell death (RCD). At the same time, severe protein oxidation can lead to the dysregulation of proteolysis, which induces either protein aggregation or superfluous protein hydrolysis. Therefore, oxidative stress contributes to the onset of age-related dysfunction. In the present review, we consider the post-translational modifications (PTMs) of proteolytic enzymes and their impact on homeostasis.

## 1. Introduction

Homeostasis is the physiological ability to maintain physical and chemical conditions throughout the internal environment of the body within tolerable limits despite external perturbations. Homeostasis is based on processes such as temperature control, osmotic equilibrium, and anabolism/catabolism balance. Anabolic pathways require energy to generate complex molecules from simpler ones. The energy and molecules for anabolism are provided by catabolism, which refers to energy production via the breakdown of macromolecules [1]. These include endogenic and exogenic proteins, lipids, carbohydrates, and nucleic acids. Macromolecule digestion is catalyzed by enzymes, which in turn are strictly regulated at different levels. These include synthesis as zymogens, compartmentalization, the requirements for specific amino acid sequences in a substrate, and the inhibition of and interaction with cofactors and signaling molecules [2].

Signaling molecules are able to transmit information within and between cells. These molecules convey signals by binding to receptors and activating second messengers. Otherwise, they directly modify target molecules, such as enzymes. These signaling molecules are represented by proteins, amino acids, nucleotides, lipids, monoamines, polyisoprenoides, metal ions, gases, reactive oxygen species (ROS), reactive nitrogen species (RNS), etc. A striking example of proteins that transmit signals is kinases. Together with nucleotides, such as adenosine triphosphate (ATP) and guanosine triphosphate (GTP), kinases regulate vital pathways [3]. Amino acids have been shown to inhibit autophagy in insulin-sensitive cells [4]. Calcium ions are ubiquitous second messengers that regulate various processes in cells by binding to EF-hand-containing proteins [5]. ROS are capable of modifying various signaling molecules and are involved in host defense processes and cell fate determination [6].

ROS and RNS are free radicals, which can be defined as atoms or molecules containing one or more unpaired electrons in a valence shell or outer orbit that are capable of independent existence [7]. The main cellular source of ROS and RNS is the mitochondrial respiratory chain. ROS and RNS are also produced in the endoplasmic reticulum, peroxisomes, and phagocytic cells with the participation of xanthine and endothelial oxidase, as well as during the autoxidation of small molecules [8]. They are also derived from exogenous sources, such as pollution, alcohol, industrial solvents, pesticides, drugs, etc. [9]. These sources are considered to be major factors that increase susceptibility to various diseases and accelerate aging [10,11].

ROS and RNS are known to cause homeostasis imbalance by inducing oxidative stress, which causes damage to lipids, proteins, and deoxyribonucleic acid (DNA) [12]. The accumulation of DNA damage leads to replicative senescence when cells are irreversibly arrested in the G1 phase of the cell cycle [13]. Since the mitochondrial respiratory chain is the main intrinsic source of ROS, oxidative-dependent damage is considered to be the major reason for mitochondrial dysfunction. This, in turn, is associated with neuronal degeneration in age-related diseases [14]. Protein oxidation disrupts the proteostasis network, which can decrease proteolysis and induce the formation of protein aggregates, such as amyloid fibrils and amorphous aggregates [15,16], related to neurodegenerative diseases and cataracts, respectively [17]. On the other hand, oxidative stress is associated with accelerated rates of the proteolytic hydrolysis of myofibrillar proteins by µ-calpain, m-calpain, and caspase 3 during disuse muscle atrophy [18].

While ROS and RNS play well-established roles in pathogenic processes, they also have important functions in regulatory and signaling processes in physiological conditions [19]. Redox signaling depends on the oxidant/antioxidant ratio and affects cell growth, differentiation, death, and other processes in cellular and tissue metabolism [20,21]. To maintain redox homeostasis, cells produce metabolites such as glutathione (GSH/GSSG) and nicotinamide adenine dinucleotide (phosphate) (NAD(H)/NADP(H)). So far, the most commonly investigated mechanism of redox regulation is mediation via the post-translational modifications (PTMs) of multiple proteins. Glutathionylation and S-nitrosylation of glyceraldehyde-3-phosphate dehydrogenase (GAPDH) influence the enzyme’s involvement in processes such as glycolysis, gene expression, and apoptosis [22]. All enzymes and several regulatory proteins of the Calvin–Benson cycle are also susceptible to redox-mediated PTMs, which influence carbon assimilation by photosynthetic organisms [23]. Several reports have revealed that proteases can also be modified by nitrosation, selfenylation, sulfinylation, glutathionylation, and sulfonation.

These modifications lead to a change in protease activity, which in turn regulates the protein content of the cell, multiple signaling pathways, cell survival, cell death, and cell and tissue homeostasis. In this review, we consider a range of mechanisms for the redox regulation of proteolytic enzymes and the impact of this regulation on homeostasis.

## 2. PTMs of Proteases Mediated by ROS and RNS

**Figure 1:**
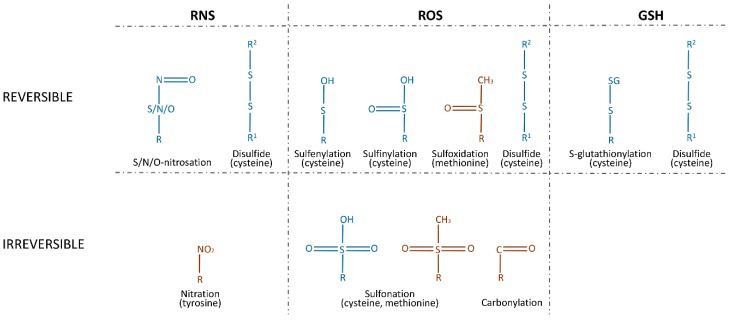
Reversible and irreversible redox-mediated post-translational modifications (PTMs) of amino acid residues. Amino acids that undergo modifications are indicated in parentheses. Modifications found for proteases are shown in blue.

A PTM is a biochemical change of a protein that takes place after translation. Most of these modifications are catalyzed by enzymes and include the specific cleavage of precursor proteins, the formation of disulfide bonds, and the covalent addition or removal of low-molecular-weight groups. A range of functional groups can be covalently added to proteins, causing phosphorylation, acetylation, adenosine monophosphate (AMP)ylation, ubiquitination, and ubiquitin (Ub)-like modifications of the target. These PTMs play a significant role in protein folding, stability, and conformation, and thus influence their functions. For many years, phosphorylation was thought to be the most prominent among these processes, but today, redox-mediated PTMs are considered to be a major class of protein PTMs. They are classified into two categories: reversible modifications and irreversible modifications. Reversible modifications are represented by sulfenylation and sulfinylation via ROS, by nitrosation via RNS, S-glutathionylation via GSH, and disulfide formation via either of them. An excessive concentration of ROS will react with reversibly sulfinylated amino acids and result in irreversible sulfonation. Nitration and carbonylation by RNS and ROS, respectively, are also irreversible (Figure 1) [24].

The increased production of hydroxyl radicals leads to the formation of lipid hydroperoxides that produce a family of α,β-unsaturated aldehydes, which react with the side chains of lysine, histidine, and cysteine residues, referred to as protein carbonylation [25]. Nitrosation and nitration are processes of incorporating NO directly or via NO donors (S-nitrosothiols (RSNOs), peroxynitrite (ONOO^–^), dinitrogen trioxide (N_2_O_3_), or nitrogen dioxide (NO_2_), etc.) as a nitroso group. S-nitrosation, O-nitrosation, and N-nitrosation were observed for the 18 amino acids [26], but only S-nitrosation has received significant attention as a PTM of proteins. Nitration, in contrast to nitrosation, is an irreversible modification. The most commonly studied example of nitration is 3-nitrotyrosine [27]. The oxidation of methionine to a sulfoxide and of cysteine to sulfenic, sulfinic, and sulfonic acids is usually mediated by H_2_O_2_, HOCl, and other ROS. With the exception of sulfonation and sulfinylation, these modifications can be reduced by dithiothreitol (DTT), thioredoxin (Trx), and nicotinamide adenine dinucleotide (NADH) [28]. Sulfinylation is reduced by the cysteine sulfinic acid reductase, sulfiredoxin (SRX) [29].Oxidized and nitrosated cysteines can be also reduced by GSH, which leads to S-glutathionylation, or by other cysteines, via the formation of disulfide bonds [30].

As can be seen, most of the PTMs of proteins are mediated by cysteine modifications. This observation can be explained by the ability of cysteines to stabilize protein conformation by disulfide bonds, which enables the cell to regulate homeostasis, for example, via GSH. To the best of our knowledge, extant research only provides evidence of redox regulation of proteolytic enzymes carried out by cysteine modifications. These include S-sulfenylation, S-sulfinylation, S-sulfonation, and the nitrosation of either the catalytic cysteine residues in cysteine proteases or the non-catalytic cysteine residues in cysteine, threonine, serine, and metalloproteases. The S-glutathionylation of non-catalytic residues in cysteine and metalloproteases was also investigated. Cysteines, which are susceptible to modification, often form—or are believed to form—disulfide bonds.

## 3. Intracellular Redox Regulation of Proteolytic Enzymes

### 3.1. Redox Regulation of Protein Turnover in Cells

The catabolism of proteins is necessary to control the quantity and quality of proteins in a cell. The digestion of unneeded and damaged proteins provides the cell with amino acids and energy. The complete hydrolysis of proteins is one of the main functions of proteolytic enzymes. There are two crucial ways that proteolysis occurs in the cell. The ubiquitine–proteasome system (UPS) uses Ub as a marker that targets cytosolic and nuclear proteins. Lysosomal proteases digest extracellular proteins and cytoplasmic organelles and proteins [31].

#### 3.1.1. Lysosomal Proteolysis

Lysosomes are the digesting organelles that mainly hydrolyze macromolecules from the secretory, endocytic, and phagocytic pathways. Lysosomes contain up to 50 enzymes, such as lipases, proteases, and nucleases. Most lysosomal proteases are ubiquitous and belong to aspartic, cysteine, and serine protease families. However, some are tissue-specific and involved in processes such as growth factor signaling, lipoprotein particle hydrolysis, infection, antigen presentation, and autophagy [32]. Lysosomal proteases are also involved in two kinds of regulated cell death (RCD): entotic and lysosome-dependent cell death [33]. Moreover, accumulating evidence indicates that lysosomal proteases are involved in extracellular matrix (ECM) hydrolysis [34,35].

A range of papain-like cysteine proteases (PLCPs), which belong to peptidase family C1 (papain family) clan CA [36], are redox-regulated lysosomal enzymes. Papain is a title protease of the family involved in the protein hydrolysis in the lysosomes of *Carica papaya* L. It is produced from the latex of *C. papaya* and plays an important role in industry [37,38]. Papain can be reversibly inhibited by the NO-mediated nitrosation of its catalytic cysteine residue 25 [39]. Cathepsin K is a collagenolytic PLCP that is mainly produced by osteoclasts and involved in bone resorption [40]. Cathepsin B is also involved in bone turnover and takes part in the processing of antigens and hormone activation [41]. Human cathepsins K and B are inhibited by a mechanism similar to the one in papain; their nitrosated residues are catalytic cysteines 25 and 29, respectively [42,43].

PLCPs are also susceptible to oxidation by H_2_O_2_. Triticain-α is a PLCP from *Triticum aestivum* L that has glutenase and collagenase activity and is believed to participate in seed maturation by digesting storage proteins during germination [44,45]. It was recently shown in our laboratory that triticain-α is inhibited by H_2_O_2_ [46]. Cathepsin D is a lysosomal aspartic protease from peptidase family A1 (pepsin family) clan AA [36]. Cathepsin D plays an important role in the hydrolysis of intracellular proteins, the activation and hydrolysis of polypeptide hormones and growth factors, the activation of enzymatic precursors, the processing of enzyme activators and inhibitors, brain antigen processing, and the regulation of programmed cell death [47]. Investigations of a rat pheochromocytoma cell line exposed to H_2_O_2_ indicated a decrease in cathepsin B activity and an increase in cathepsin D activity. However, the mechanisms of these processes are unknown [48]. Cathepsin S is a PLCP expressed predominantly in immune cells and is crucial for the processing of the invariant chain in antigen-presenting cells [49]. Human cathepsins K and S are inhibited by H_2_O_2_ via the PTMs of their catalytic cysteines. Cathepsin K is mainly oxidized to irreversible sulfonic acid in a time- and dose-dependent manner [50], whereas procathepsin S is oxidized to reversible sulfenic acid, which inhibits its autocatalytic maturation [51]. Cathepsin S oxidation is reversed by cysteine or GSH [51]. Cathepsin L is a PLCP that, apart from protein turnover in lysosomes, is involved in H3-histone and prohormone processing in the nucleus and secretory vesicles, respectively [49]. It was shown that oxidative stress suppresses the autocatalysis of procathepsin L [52]. The treatment of human fibroblasts with 1-methylnaphthalene-4-propionateendoperoxide (MNPE) and naphthalene-1,4-dipropionate endoperoxide (NDPE), which generate singlet oxygen, inhibits cathepsins B, L, and S. Singlet oxygen also inhibits papain in vitro. However, the mechanism of this action is ambiguous [53]. Cathepsin S and papain can be inhibited by ROS indirectly via the irreversible glycation of the active site by carbonyls that accumulate during oxidative stress [54,55].

Since the catalytic cysteines in PLCPs can be oxidized either reversibly or irreversibly, it was suggested that reversible PTMs protect the enzymes from irreversible modifications under conditions of severe oxidative stress [56]. Interestingly, cathepsin D is the only lysosomal aspartic protease that is susceptible to redox regulation and the only lysosomal protease investigated so far whose activity is increased by ROS. This observation provides a direction for future research into the mechanisms of aspartic protease redox regulation.

#### 3.1.2. Ubiquitine-Proteasome System

The UPS consists of multiple enzymes and regulatory proteins that, unlike lysosomal enzymes, mainly digest the unnecessary and misfolded proteins involved in the cell cycle, transcription, and growth. Digestion is provided by the proteasome, which is a multi-subunit threonine protease complex subjected to alterations derived from oxidative stress. Proteasomal subunits are susceptible to carbonylation, proteasomal glycoxidation, and modification with lipid peroxidation products. These PTMs lead to a decrease in proteasome activity, although most of them target non-proteolytic subunits. The 20S core proteasome contains only three catalytic subunits, β1, β2, and β5, which belong to the peptidase family T1 (proteasome family), clan PB [36]. Two of them, β2 and β5, along with the β4 and β6 subunits, are subjected to the glycoxidation and glycation promoted by oxidative stress. This PTM inhibits proteasome activity. However, the mechanisms of this process remain ambiguous [57]. On the other hand, two S-glutathionylated cysteine residues in the α5 subunits of 20S in yeast proteasomes mediate the opening of the annulus, which increases the activity of the proteasome [58].

The proteasome is also susceptible to indirect redox regulation. It was shown that oxidized proteins are hydrolyzed by the 20S proteasome independent of both the regulatory 19S complex and Ubsince adding ATP to the cell lysate had no effect or even decreased proteasomal hydrolysis [59,60,61]. Oxidative stress influences the 26S proteasome and the ubiquitinating machinery more strongly than its 20S core [62,63]. Thus, 20S proteasomal hydrolysis is unaffected by H_2_O_2_ concentration up to 5mM, while ATP-dependent hydrolysis via the 26S proteasome begins to decline at 400 µM and is completely abolished at 1 mM [64,65]. Hence, the 26S proteasome disassembles on 20S and 19S particles under oxidative stress conditions [66].On the other hand, it was shown that carbonyl-containing proteins, a direct measure of protein oxidation, are selectively removed by ubiquitinating machinery, and, therefore, some oxidized proteins might require Ub-dependent 26S proteasome hydrolysis [67,68].

The ubiquitination of proteins depends on two kinds of enzymes. The E3 ubiquitin ligase conjugates Ub to a lysine residue of a target protein, which serves to direct the protein for hydrolysis by the proteasome. Conversely, deubiquitinating enzymes (DUBs) eliminate Ub from the substrates and inhibit proteasomal hydrolysis.

DUBs include the cysteine proteases from peptidase families C12 (Ub C-terminal hydrolase family), C19 (Ub-specific protease family), C64, and C65 of clan CA, as well as the metallopeptidases from family M67 of clan MP [36]. Cysteine DUBs are susceptible to redox regulation since they contain a low-pKa catalytic cysteine residue. It was reported that Ub-specific peptidase 1 (USP1), a DUB involved in the DNA damage response pathways, is reversibly inactivated by the sulfenylation of catalytic cysteine [69]. The inhibition of USP1 activity after oxidative DNA damage leads to the time-dependent accumulation of monoubiquitinated proliferating cell nuclear antigen (PCNA). This process enables a gap repair of oxidized DNA lesions during the S phase [69]. Multiple ovarian tumor DUBs were also shown to undergo cysteine oxidation upon H_2_O_2_ treatment [70]. Transient RSOH can then be stabilized by the formation of hydrogen bonds with the highly conserved residues in the loop preceding catalytic cysteine. DUBs exhibit distinct levels of sensitivity to oxidation depending on various ranges of catalytic activation, in which the conformationally inactive enzyme can be less susceptible to oxidation. For example, USP7, preincubated with Ub, which behaves as an allosteric activator, is more susceptible to ROS [71]. Therefore, the reversible inactivation of DUBs mediated by ROS may regulate the ubiquitination status of many signaling molecules [71] and promote hydrolysis by the proteasome.

The contradictory redox regulation of UPS can be explained by its dose dependence on ROS. However, more research must be conducted to validate this hypothesis. To the best of our knowledge, the redox regulation of proteasomes is the only example of the redox regulation of threonine proteases. It would be interesting to see whether other threonine proteases with similar structures are susceptible to redox regulation.

#### 3.1.3. PTMs Induced by Proteases

Apart from catabolic processes, cellular proteolytic enzymes are involved in various regulatory pathways. Deubiquitinating proteases can eliminate Ub-mediated PTMs. Several Ub-like proteins, such as MoaD, ThiS, SUMO, NEDD8, ATG8, and ATG12, can be conjugated by ligases and digested by proteases from the target protein [72]. The most commonly investigated example is the small Ub-like modifier (SUMO), which is necessary for regulation of the cell cycle and apoptosis [73]. This is responsible for processing the full-length SUMO into its mature form and deconjugating SUMO from targeted proteins [74].

SUMO proteases belong to the peptidase family C48 (Ulp1endopeptidase family) of clan CE, C97 of clan CP, and C98 of clan CA [36]. However, redox regulation was only observed for family C48. H_2_O_2_ treatment induces the reversible formation of an intermolecular disulfide bond between catalytic and non-catalytic cysteines in the yeast SUMO Ub-like-specific protease 1(ULP1) and in its human equivalent, sentrin-specific protease 1 (SENP1) [75].At the same time, sulfonation of the active site causes an irreversible inhibitory effect [75]. Therefore, disulfide bonds can protect the proteases from more severe oxidation.

In summary, there is no doubt that proteolysis inside the cell is redox-regulated. However, the redox regulation of lysosomal hydrolysis has been mainly described for cysteine proteases, although the regulation of aspartic and serine proteases is also important for protein turnover and intracellular homeostasis. The redox regulation of deubiquitinating and deSUMOylating proteases also requires more precise research into its influence on different signaling pathways. It would be interesting to investigate the redox regulation of DUB metallopeptidases. The molecular mechanisms of the redox regulation of the proteasome are quite ambiguous. However, this unique example of redox regulation of threonine protease provides a guideline for future investigations of other proteases from this group.

### 3.2. Redox Regulation of RCD

Proteastasis is maintained by the UPS, which digests misfolded and aggregated proteins. However, continuous stress increases the amount of harmful proteins, and aging decreases the number of proteasomes. These lead to overwhelming of the UPS, proteostasis collapse, and cell death. ROS and RNS themselves can cause either accidental cell death (ACD) or RCD. In contrast with ACD, the instant and catastrophic collapse of cells exposed to severe physical, chemical, or mechanical insults, RCD depends on molecular machinery and, therefore, can be modulated. RCD also plays a major role in development, tissue homeostasis, inflammation, immunity, and multiple pathophysiological conditions. RCD can be subdivided into 12 distinct modes: intrinsic apoptosis, extrinsic apoptosis, mitochondrial permeability transition (MPT)-driven necrosis, necroptosis, ferroptosis, pyroptosis, parthanatos, entotic cell death, neutrophil extracellular traps (NET)otic cell death, lysosome-dependent cell death, autophagy-dependent cell death, and immunogenic cell death [33].

#### 3.2.1. Initiation of RCD

RCD can be induced either by physiological programs for development or tissue turnover or by intense and prolonged perturbations of the microenvironment. The main function of the mitochondria is ATP production for cell survival and other vital functions [76]. However, mitochondria also act as central executioners in different kinds of RCD, such as apoptosis, MPT-driven necrosis, necroptosis, and parthanatos [33].

Mitochondria-mediated cell death can be induced by DNA damage, intracellular Ca^2+^ overload, and oxidative or ER stress. Calpains (CAPNs) are calcium-dependent cysteine proteases from the peptidase family C2 (calpain family) of clan CA [36], which plays an important pro-death role after Ca^2+^ overload. In particular, CAPNs cleave caspase 3, apoptosis-inducing factor(AIF), and Bid, and induce cytochrome *c* release [77].

ROS can initiate CAPN activation indirectly by increasing intracellular Ca^2+^ or directly by inducing oxidative modifications of the proteases themselves [78,79,80,81]. Moreover, ROS-mediated modifications can affect the susceptibility of different proteins to CAPN-mediated cleavage [18,82]. The inactivation of NADPH oxidase attenuates µ-CAPN activity and decreases apoptosis in pulmonary microvascular endothelial cells (PMECs) during septic plasma stimulation. Therefore, targeting µ-CAPN and calpastatin may prevent endothelial injury in sepsis [83]. It was also shown that administration of an antioxidant, 6-hydroxy-2,5,7,8-tetramethylchroman-2-carboxylic acid (Trolox), prevented the activation of µ-CAPN and caspase 3, indicating that oxidative stress is required for the activation of the major proteolytic pathway underlying muscle atrophy [84]. The sustained activation of CAPNs in the platelets from diabetic patients was found to be a consequence of the redox-mediated dysregulation of intracellular Ca^2+^ homeostasis [81].

The activity and autolysis of μ-CAPN are reversibly inhibited by H_2_O_2_. Tandem mass spectrometry (MS) analysis revealed disulfide bond formation between the catalytic and non-catalytic cysteines [85]. In vitro studies revealed that m-CAPN is susceptible to inhibition via the singlet oxygen generated by MNPE [86]. The activities of CAPNs are also redox-regulated by S-nitrosation. Indeed, m-CAPN isolated from either skeletal muscle or neutrophils can be S-nitrosated and inactivated by sodium nitroprusside (SNP). This effect can be reversed by DTT, suggesting the modification of a cysteine residue [87]. More recently, in vitro studies confirmed the effect of S-nitrosation on the autolysis and activity of µ-CAPN and identified the S-nitrosated cysteine residues as cysteine 49, cysteine 351, cysteine 384, and cysteine 592 in µ-CAPN and cysteine 142 in a small regulatory subunit 1 (CAPN4) [88]. µ-CAPN is less sensitive to modification by NO at a neutral pH, and inactivation could be only observed in acidic conditions [80]. Based on these observations, it was suggested that NO may regulate CAPN activity depending on pH fluctuations in contracting cells or pathological conditions [87]. The role of NO in the regulation of CAPN activity has since been confirmed, as well as the age-related decrease in the expression of neuronal nitric oxide synthase (NOS) is correlated with a decrease in CAPN S-nitrosation. This, in turn, leads to CAPN activation and myofibril hydrolysis [89]. The link between NO and CAPN was also supported by the fact that NOS inhibition promoted µ-CAPN autolysis, resulting in the hydrolysis of titin and nebulin during post-mortem aging [90]. Therefore, the S-nitrosation of CAPNs was associated with protection against myocardial ischemia/reperfusion injury [91].

CAPN activity is also redox-regulated indirectly since the recognition of oxidized proteins by CAPNs is enhanced [18]. For instance, the Ca^2+^-induced production of ROS leads to AIF carbonylation and accelerated cleavage by µ-CAPN [92], processes that can be blocked by antioxidants [82]. This physical interaction and subsequent hydrolysis of AIF by µ-CAPNs may be redox-dependent. This follows from the fact that the recognition and proteolysis of protein targets by µ-CAPN are expedited by PEST motifs, which are known to be redox-sensitive [93,94]. Moreover, the oxidation of myofibrillar proteins enhances their susceptibility to proteolytic hydrolysis by CAPNs, connecting oxidative stress with accelerated myofibrillar proteolysis during disuse muscle atrophy [18]. However, oxidative PTMs of the muscle intermediate filament desmin decrease susceptibility to CAPNs and simultaneously increase caspase 3 and 6 mediated hydrolysis [95].

Mitochondria-mediated RCD can also be induced by a protein quality control system. Proteostasis requires a critical oversight of misfolded or aggregated proteins, which can be either refolded or hydrolyzed. Cells have developed several quality-control mechanisms, such as chaperones, the unfolded protein response (UPR), autophagy, endoplasmic reticulum associated protein hydrolysis (ERAD), and proteasomes. However, when the capacity of the protein homeostasis network is overwhelmed, the cellular response switches from pro-survival to pro-death.

The mitochondrial inner membrane (IM) Oma1 is a metalloproteinase from the peptidase family M48 (Ste24 endopeptidase family) of clan MA [36]. It is a central controller of mitochondrial membrane homeostasis and dynamics [96,97,98]. Oma1 maintains a semi-oxidized state across different organisms, so the activity and stability of the Oma1 oligomeric complex is susceptible to redox regulation. It is mediated by two highly conserved inner membrane space-exposed cysteine residues, cysteine 272 and cysteine 332, which can form disulfide bonds. These bonds maintain the structure of the Oma1 complex [99]. Data indicate that only thiol-containing reductants decrease the proteolytic activity of Oma1. Moreover, the proteases lacking these cysteine residues are less sensitive to reduction [100]. A similar mechanism was found in serine enterobacterial Lon protease, which has been proposed to function as a switch to optimize Lon’s proteolytic activity depending on the redox environment [101]. ROS and RNS often induce the misfolding and aggregation of proteins. Hence, stabilization of the Oma1 structure under oxidative conditions supports proteostasis control under stress.

To summarize, the mechanisms of the redox regulation of cysteine proteases involved in RCD induction have been thoroughly investigated, although the line between cell death induction and cell death execution remains ambiguous. Moreover, other groups of proteases are involved in cell death induction. For instance, granzymes, which belong to the peptidase family S1 (chymotrypsin family) of clan PA [36], can eliminate virally infected and malignant cells [102]. Unraveling the mechanisms of redox regulation of this group of proteases is still needed.

#### 3.2.2. Plant RCD

Different methods of inducing cell death lead to different kinds of RCD. Plant RCD is usually divided into two types: developmental and pathogen-triggered (dRCD and pRCD) [103,104]. ROS and calcium initiate execution directly in dRCD or via salicylic acid (SA) production in pRCD. Proteolytic enzymes, such as metacaspases, phytaspases, saspases, UPS, and vacuolar processing enzymes (VPEs), which have caspase-like activity, act as executors [105]. Autophagy is also involved in plant RCD as an effector of pPCD and as a corpse clearance mechanism during dPCD [106].

Although ROS and proteases were shown to take part in plant RCD [107], only one mechanism of redox regulation is currently known for the proteolytic enzymes involved in plant RCD. Metacaspase 5 from the diatom *Phaeodactylum tricornutum* (PtMC5) most likely belongs to the peptidase family C14 (caspase family) of clan CD [36]. Its role in algae is still elusive. However, it was reported that PtMC5 contains potential disulfide-forming cysteines, which are conserved in diatom type III metacaspases. The mutagenesis of these residues decreases PtMC5 activity, indicating redox regulation. It was suggested that ROS oxidize these cysteines, inducing disulfide bond formation, thereby increasing PtMC5 activity. This PTM leads to the execution of an RCD pathway [108]. Hence, this mechanism could be a link between ROS accumulation during the RCD induction phase and protease activation to start the execution phase [109].

#### 3.2.3. Apoptosis

Apoptosis is the most commonly investigated kind of RCD promoted by caspases. Caspases are peptidases from family C14 (caspase family) of clan CD [36] and play essential roles in the initiation and execution phases of apoptosis. Caspases 2, 8, 9, and 10 mediate the initiation phase, and caspases 3, 6, and 7 mediate the execution phase of apoptosis. They serve as primary effectors of the proteolytic destruction of most cellular structures, including the cytoskeleton, cell junctions, mitochondria, endoplasmic reticulum, Golgi apparatus, and nucleus [110]. Caspases are optionally involved in the induction and execution stages of lysosome-dependent cell death and pyroptosis, as well [33]. However, there are no reports of redox regulation in these processes. 

There is some evidence that caspase activity is controlled by post-translational modifications, such as oxidation, nitrosation, and glutathionylation, of catalytic site cysteine residues [111]. The H_2_O_2_-mediated redox-dependent intramitochondrial autoactivation of caspase 9 has been demonstrated in U937 cells where procaspase 9 dimerization was induced by thiol–disulfide bond formation. This process was inhibited by Trx [112]. Procaspase 9 is activated in the preapoptotic phase before cytochrome c release and, therefore, may amplify the proapoptotic effect of cytochrome c [112]. On the other hand, there are reports that H_2_O_2_ inactivates caspase 9 through the oxidation of cysteine residue in the active site of the enzyme via a process catalyzed by iron [113]. H_2_O_2_ also induces the reversible inactivation of caspases 3 and 8 by oxidizing their catalytic site cysteines [114]. In vitro studies showed that singlet oxygen derived from MNPE irreversibly inhibited cysteine in the active site of caspase 3 [86].

Low levels of NO have been shown to exert antiapoptotic effects via the S-nitrosation of a single cysteine residue at the catalytic sites of caspases [115,116]. Seven members of the caspase family have been shown to be inhibited by this mechanism [117]. The cellular sites of caspase nitrosation/denitrosation have not been fully investigated, but mitochondria are reportedly key locations for S-nitrosation reactions [118]. Studies on inhuman umbilical vein endothelial cells using electron spin resonance spectroscopy showed that the S-nitrosation of cysteine 163 of caspase 3 inhibits protease and prevents the caspase 3 mediated apoptotic cascade [119]. In hepatocytes, NO blocks Bid activation through the S-nitrosation of caspase 8 and prevents tumor necrosis factor alpha (TNFα)-induced mitochondrial apoptosis signaling [120,121,122]. Additionally, NO donors were found to inhibit the proper assembly of the Apaf-1/caspase 9 apoptosome complex and caspase 9 activation [120,121,122]. At the same time, it was reported that denitrosation is proapoptotic. For instance, denitrosation of procaspase 3 and procaspase 9 was shown to be associated with proteolytic enzyme activation in Fas-mediated [123] and cytokine-induced [124] apoptosis.

Apart from ROS and RNS, GSH takes part in the redox regulation of caspase activity through S-glutathionylation. The S-glutathionylation of caspase 3 increases the stability of the protease and decreases its accessibility for proteolytic cleavage. Moreover, the S-glutathionylation of catalytic cysteine in caspase 3 results in the inhibition of caspase activation and activity [125]. Altogether, this leads to apoptosis resistance. Conversely, it was demonstrated that the deglutathionylation of caspase 3 increases caspase 3 activity and TNFα-induced endothelial cell apoptosis [126]. The reversible glutathionylation of caspase 3 is mediated by TNFα-induced glutaredoxin during TNFα-mediated cell apoptosis [127].

Since caspases are cysteine proteases, they are highly sensitive to inhibition by the PTMs of catalytic cysteine. At the same time, ROS can induce their activation. It will be necessary to determine how these contradictory mechanisms coordinate during apoptosis. Additionally, it would be helpful to determine the mechanisms of redox regulation for the other proteases that promote apoptosis. For instance, it was shown that proteasome inhibitors induce oxidative stress, which in turn leads to apoptosis [128].

#### 3.2.4. Autophagy

In contrast to apoptosis, the autophagy machinery is associated with cell survival in selective autophagy. This process requires the formation of double-membrane vesicles and an autophagosome for the hydrolysis of dysfunctional organelles and misfolded or aggregated proteins. This provides cells with molecular building blocks during periods of nutrient deprivation [129]. Autophagy-dependent cell death is a pathway for cellular self-digestion. It is mediated by the formation of autophagosomes, which confine cellular components and deliver them to the lysosomes for hydrolysis. This autophagy machinery is also used for entotic cell death, which is a form of mammalian cell cannibalism, and immunogenic cell death, which is necessary to activate an adaptive immune response specific to antigens expressed by dying cells [33].

Autophagy is mediated by dozens of autophagy-related (ATG) proteins. The 15 core genes encoding ATG proteins in yeast are conserved in mammals, indicating that autophagy is an evolutionarily conserved process [130]. Among them, ATG4 is the sole protease from peptidase family C54 (Aut2 peptidase family) of clan CA [36]. It regulates autophagy through the processing and deconjugating of the ATG8/LC3 protein [131]. The processing of ATG8/LC3 by ATG4 exposes a glycine residue at the C-terminus of ATG8/LC3 for further conjugation with phosphatidylethanolamine (PE). This is crucial for autophagosome formation. The deconjugation of ATG8/LC3-PE by ATG4 recycles ATG8/LC3 for the next round of the conjugation reactions. Therefore, ATG4 can act as both a conjugating and deconjugating enzyme, and the fine regulation of these two activities is essential for the normal function of autophagy.

At present, ATG4 is the only ATG protein whose activity has been shown to be redox regulated [132,133]. In humans, H_2_O_2_ inhibits ATG4A and ATG4B through cysteine 81 oxidation, which results in either the shielding of nearby catalytic cysteine 77 or the formation of a disulfide bond between cysteines 77 and 81 [132]. The same mechanism of ATG4 redox regulation was also found in other organisms. Different assays showed that the redox regulation of ATG4 in *Saccharomyces cerevisiae* is mediated by a disulfide bond between cysteines 338 and 394. This disulfide has low redox potential and is very efficiently disrupted by Trx reduction [134]. If these cysteines are replaced, ATG4 activity increases, which leads to the accumulation of ATG8-PE. The redox regulation of yeast ATG4 may, therefore, be crucial for controlling the equilibrium between free ATG8 and ATG8-PE and, thereby, the initiation of phagophore assembly site formation [134]. The incubation of prereduced ATG4 protein with S-nitrosoglutathione (GSNO) results in ATG4 activity inhibition. This is completely reversed by DTT but not by another RSNO-specific electron donor, such as ascorbate, again supporting the formation of a disulfide bond. Since the disulfide bond is situated near the active site, it may restrain access to the catalytic cysteine and prevent ATG8 binding [134]. A similar mechanism was also shown in other organisms, such as green alga *Chlamydomonas reinhardtii*. *C. reinhardtii* ATG4 has a single disulfide bond with low redox potential. Its reduction by NADPH and Trx regulates the activity of the protease. Norflurazon inhibits carotenoid biosynthesis, generates reactive oxygen species, and triggers autophagy in *C. reinhardtii*. It promotes the oxidation and aggregation of ATG4. The inhibition of ATG4 under stress conditions leads to the lipidation of ATG8 and thus autophagy progression in *C. reinhardtii* [135]. This mode of regulation was also shown in other Trx-regulated enzymes [136,137]. The activity of *Arabidopsis thaliana* ATG4a and ATG4b is also reversibly inhibited by oxidation [133]. However, the underlying molecular mechanisms have not yet been reported.

Taken together, the data indicate that ATG4 is inactive under stress conditions to prevent delipidation of ATG8/LC3 at the site of autophagosome formation. However, since ATG4 is also crucial for ATG8/LC3 lipidation, it is important to investigate how the redox regulation of ATG4 depends on time and space. Lysosomal proteases, which were described previously, participate directly in the execution of autophagy. Their dysregulation by oxidative stress may inhibit autophagy and thus prevent cell survival.

To conclude, the redox regulation of proteolytic enzymes involved in RCD is important. The cell response to oxidative stress should be dose-dependent. Low concentrations of ROS ought to induce cell-survival pathways. However, RCD is necessary in excessive oxidation to avoid ACD, which often causes inflammation in nearby cells. Hence, redox-sensitive proteolytic enzymes act as indicators of the amount of ROS, thereby inducing appropriate signaling pathways. The redox regulation of apoptosis and autophagy have investigated the most thoroughly in the literature. Therefore, it will be necessary to conduct research on the redox regulation of proteolytic enzymes for other kinds of RCD, such as NETotic cell death, which is believed to be an ROS-dependent modality of RCD restricted to cells of hematopoietic derivation [33].

### 3.3. Redox Regulation of Proteases Involved in Inflammation

Cell death is closely related to inflammation since it can either induce or be induced by inflammation. Inflammation occurs in response to various factors, such as infection, tissue injury, or cardiac infarction [138]. Infectious inflammation is promoted to protect host tissues from invading microorganisms, such as bacteria and viruses. However, many pathogens have evolved to subvert host cellular processes, including autophagy machinery and caspases, to further propagate infection [139]. This mechanism often involves proteases of the pathogen; hence, their inhibition can defend the host organism.

A range of viral and parasitic proteases are inhibited through NO-mediated modifications of catalytic cysteine. Different NO donors dose-dependently inhibit PLCP (peptidase family C1 (papain family), clan CA [36]) falcipain from *Plasmodium falciparum* trophozoite extract and cruzipain from *Trypanosoma cruzi*. Falcipain has hemoglobinase activity and takes part in chloroquine-mediated cell death [140]. Cruzipain is involved in parasite nutrition, cell invasion, and immune response evasion [141]. It is likely that these effects are attributable to the S-nitrosation of the cysteine 25 catalytic residues [142,143]. The reversible inactivation of cysteine proteases by nitrosation and oxidation may protect the proteases against more harmful oxidants [56]. Coxsackie viral proteases 2A and 3C belong to the peptidase family C3 (picornain family) of clan PA [36]. They process viral polyprotein and hydrolyze host cell protein to facilitate viral replication [144]. NO donors inhibit them through the S-nitrosation of catalytic cysteines 110 and 147, respectively. These NO-mediated inhibitory effects are dose-dependent and reversed by DTT [145,146].

Over the course of their life cycle, pathogens produce specific molecules known as pathogen-associated molecular patterns (PAMPs). The recognition of PAMPs, as well as damage-associated molecular patterns (DAMPs), results in the assembly of the inflammasome. Inflammasomes are multi-molecular protein complexes that are responsible for the inflammation in immune cells through the activation of caspase 1 (family C14 (caspase family) of clan CD), which, in turn, processes the precursors of pro-inflammatoryinterleukin-1β(IL-1β), IL-18, and other cytokines [147].

NACHT, LRR, and PYD domain-containing protein 3 (NLRP3) inflammasome formation occurs independent of the UPR but requires the production of ROS and K^+^ efflux [148], which suggests the involvement of redox regulation in this process. Indeed, the crystal structure of NLRP3 contains a highly conserved disulfide bond between cysteines 8 and 108 connecting the PYD domain and the nucleotide-binding site domain, which are highly sensitive to altered redox states [149]. The disulfide bond is strictly conserved, indicating that it plays a crucial redox role in the regulation of NLRP3 [150]. Since NLRP3 formation is necessary for caspase 1 activation, this mechanism represents the indirect redox regulation of proteolytic enzyme.

Therefore, the currently understood mechanisms of the redox regulation of proteolytic enzymes involved in inflammation relate to cell protection. ROS and RNS inhibit the proteolytic apparatus of the invading pathogens and promote inflammasome formation. However, to the best of our knowledge, caspase 1 is the only host protease involved in inflammation that has been shown to be redox regulated. It is necessary to investigate the redox regulation of other inflammation-related proteases.

## 4. Extracellular Redox Regulation of Proteolytic Enzymes 

### 4.1. Redox Regulation of Proteases Involved in Inflammation

If the immune system exceeds its capacity, inflammation progresses to a chronic state, which is associated with many diseases, such as allergies, coeliac disease, hepatitis, reperfusion injury, and pulmonary hypertension. These diseases are usually characterized by the excessive production of ROS by inflammatory cells. Superoxide dismutase 3 (SOD3) is an extracellular antioxidant enzyme that converts superoxide radicals into hydrogen peroxide and oxygen. It is susceptible to PTMs by neutrophil-derived HOCl at the inflammatory sites of lung injury. The destabilization of a protein’s structure via the oxidation of the N-terminal region and the formation of intermolecular cross linking increase the protein’s susceptibility to proteolysis by neutrophil-secreted proteases [151]. Apparently, SOD3 is necessary to protect cells from oxidative stress in healthy tissues. However, during inflammation, SOD3 is inhibited for ROS to promote inflammasome formation and cytokine secretion.

Inflammation can be induced by pathogen invasion. In contrast to intracellular pathogens, extracellular ones have to avoid phagocytosis and thus promote extracellular multiplication. Virulence requires proteolysis over the whole infection cycle of the pathogen to facilitate penetration and dissemination. Hence, they enable colonization of the host, combating its defense mechanisms [152]. However, there have been few investigations into the redox regulation mechanisms of proteases produced by extracellular pathogens.

### 4.2. Redox Regulation of Proteases Involved in Tissue Remodelling

There is increasing evidence for the excessive activity of matrix metalloproteinases (MMPs) in inflammatory diseases and cancer [153]. MMPs belong to the family M10, clan MA [36]. They are represented by zinc-containing enzymes that play a key role in ECM remodeling—the development and maintenance of tissues and organs, as well as the overall morphology of an organism [154,155]. They are also involved in bone remodeling, angiogenesis, immunity, wound healing, and malignant tumor progression [156].

It was shown that a variety of oxidants regulate MMP activity in different inflammatory conditions [157]. The regulation of proteases is carried out allosterically through modifications of cysteine residues, which coordinate metal ions in the catalytic sites of MMPs. PRCGVPDVA is a highly conserved sequence in the prodomain of matrilysin (MMP7). This sequence contains the cysteine that coordinates catalytic Zn^2+^ and thereby promotes the inactivity of the enzyme. It was shown that HOCl, but not H_2_O_2_, converts the thiol in the cysteine residue of pro-MMP7 into sulfinic acid, which leads to the disruption of zinc coordination [157]. The activation of other MMPs engages in similar redox regulation [158]. Pro-MMP2 is also susceptible to redox modifications, such as S-glutathionylation and S-nitrosation [159]. It was shown that peroxynitrite with GSH causes S-glutathionylation of the propeptide cysteine residue, which leads to activation of the enzyme. MMP1, MMP8, and MMP9 have the same mechanism of activation [160]. The activation of MMPs by ROS and RNS is associated with a range of disorders and diseases. Therefore, the redox regulation of MMPs represents redox dysregulation in the case of oxidative stress [161].

MMPs and cathepsins are also involved in tissue turnover in the tumor microenvironment [34,35,49,162,163,164,165]. Although the mechanisms of the redox regulation of proteases in the ECM of cancer are quite ambiguous, there are some prospective directions for research in this field. ROS regulate lysosomal exocytosis in a dose-dependent manner. While moderate levels of ROS stimulate lysosomal exocytosis and thereby increase the amount of proteases in the ECM, pathological levels of ROS inhibit the process [166]. As we already mentioned, cysteine cathepsins are inhibited by ROS and RNS. This counteracts the superfluous levels of ROS in the tumor microenvironment [167]. On the other hand, H_2_O_2_ increases the activity of aspartic cathepsin D in pheochromocytoma cells (PC12) [48], which possibly explains the excessive protease activity in the tumor ECM. The pathological activities of cathepsins in the tumor microenvironment promote tumor growth, angiogenesis, and metastasis. Understanding the mechanisms of redox regulation in malignant tumors can support the development of novel therapies for cancer.

### 4.3. Redox Regulation of Proteases Involved in Shedding

The regulation of complex processes such as inflammation and tissue remodeling requires intracellular signaling. Signal molecules can be emitted from the cell by vesicular transport or ectodomain shedding. Ectodomain shedding releases a variety of proteins, such as cytokines, receptors, and enzymes, which are anchored in the membrane [168].

A disintegrin and metalloprotease domain 17 (ADAM17) is a transmembrane metalloproteinase of peptidase family M12 (astacin/adamalysin family), clan MA [36], which activates the precursors of membrane-anchored proteins by ectodomain shedding [169]. Redox agents and oxidoreductase activity target the sulfhydryl groups of ADAM17. Two vicinal cysteine sulfhydryl motifs (C522XXC and C600XXC) contain redox-sensitive sites in the disintegrin/cysteine-rich region of ADAM17. Oxidation by H_2_O_2_ induces disulfide bond formation and switches ADAM17 conformation from less active to fully active. Conversely, DTT mediates the reversible inhibition of the enzyme. This thiol-disulfide conversion within the extracellular portion of ADAM17 may be crucial for the ectodomain shedding of L-selectin upon neutrophil activation [170]. On the other hand, redox modification of ADAM17 may provide a general activation mechanism. Others have reported that treating cells with sulfhydryl-modifying agents induces the ectodomain shedding of TNFα receptors [171,172]. L-selectin and TNFα are important for immune response and inflammation, so their activation by redox-sensitive ADAM17 may provide a protective mechanism to counter oxidative stress.

### 4.4. Redox Regulation of Proteases Involved in Digestion

Inflammation is usually initiated as a result of a hypersensitive immune reaction to exogenic molecules. The proteolytic digestion of the exogenic proteins in multicellular organisms starts in the gastrointestinal tract. Pepsin is an aspartic protease of peptidase family A1 (pepsin family), clan AA [36], responsible for peptide bond digestion in the acidic conditions of the stomach. Despite the importance of this enzyme for the hydrolysis of proteins, there are no reports on its susceptibility to redox regulation. Trypsin and chymotrypsin are serine proteases that belong to peptidase family S1 (chymotrypsin family), clan PA [36]. They are produced by the pancreas and are then activated in the small intestine, where they play a principal role in protein digestion. The singlet oxygen derived from MNPE irreversibly inhibits bovine trypsin and chymotrypsin in vitro. The mechanism of this process has not been revealed, but histidine and serine residues of a catalytic triad were suggested to be possible targets of oxidation [86].

The redox regulation of homeostasis outside the cell can either increase the damaging effect of oxidative stress or induce protective mechanisms during inflammation. An investigation into the new redox-mediated PTMs of proteolytic enzymes is necessary to obtain a deeper understanding of the interactions among these processes. This should help in treating many inflammatory disorders. It would also be useful to compare these interactions with the redox regulation mechanisms of proteases in healthy tissues and to conduct investigations of the underlying mechanisms of trypsin and chymotrypsin inhibition.

## 5. Directions for Future Research

**Figure 2:**
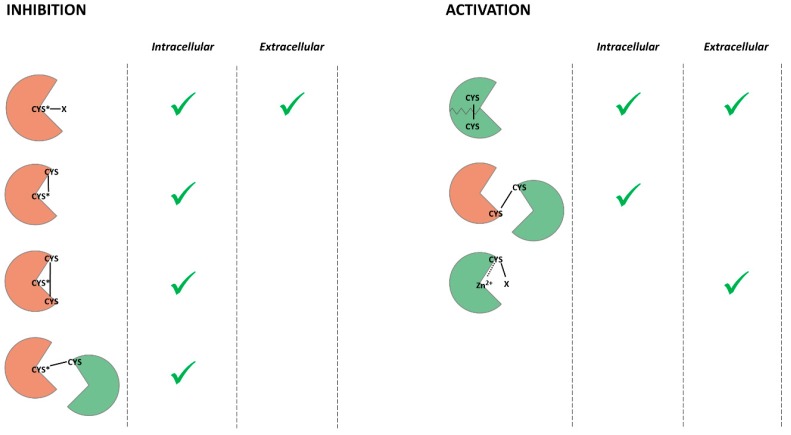
Mechanisms of the redox-mediated modifications of proteolytic enzymes resulting in the inhibition or activation of proteases. Inactive enzymes are indicated in red, and active enzymes are in green. Intracellular or extracellular localizations are indicated by green checkmarks.X—either SNO, SOH, SO_2_H, SSG (reversible PTM), or SO_3_H (irreversible PTM); CYS—cysteine; CYS *—catalytic cysteine; Zn^2+^—catalytic zink ion; solid line—the covalent bond; dashed line—the broken covalent bond.

Herein, we discussed recent discoveries in cellular and tissue homeostasis and redox regulation. ROS and RNS can cause oxidative stress and induce multiple age-related diseases. However, accumulating data indicate that ROS and RNS act as secondary messengers in the signaling cascades involved in the determination of cell fate. The redox regulation of proteolytic enzymes leads to shifts in cell proteostasis. ROS, RNS, and GSH modify the intracellular and intercellular proteases regulating proteolytic activity. Redox-mediated PTMs can inhibit or activate proteases, promote autocatalysis or aggregation, and maintain the structure of the enzyme (Figure 2).

Considering all of the findings supporting the redox regulation of proteolytic enzymes, it is clear that this research area is quite ambiguous. Firstly, much of the research lacks either data on the molecular mechanisms of PTMs by ROS and RNS or data concerning the homeostatic impact of the observed modifications. Secondly, some proteases are reported to be inhibited and activated by the same compounds. However, there is no work investigating both effects at once. Thirdly, to the best of our knowledge, the only redox-mediated modifications of proteases that have yet been investigated are modifications of cysteine amino acid residues. Hence, studies on the redox regulation of cysteine proteolytic enzymes prevail over studies on other families of proteases.

Redox-mediated post-translational modifications of cysteines can either inhibit or activate proteolytic enzymes (Figure 2). Catalytic cysteine thiol is necessary for protein digestion by cysteine proteases, so the PTMs of catalytic cysteine inhibit this group of proteolytic enzymes. Cysteine thiols are also able to coordinate metal ions, so cysteines in a prodomain of metalloprotease inhibit the activity of the enzyme. Therefore, the PTMs of these cysteines lead to the activation of the enzyme. Cysteine disulfides stabilize the tertiary structure of many proteins and, in some cases, are the only type of interaction maintaining the protein fold. Hence, their destruction by reducing compounds causes a decrease or inhibition of catalytic activity. However, if the disulfide bond localizes near catalytic amino acid residue, it can prevent substrate binding.

The amount of data concerning the redox regulation of proteolytic enzymes has dramatically increased during the last 20 years. At present, there is no doubt that ROS and RNS have both physiological and pathological functions. However, researchers still face many challenges. The line between the damaging oxidation of proteins over the course of oxidative stress and redox regulation mediated by PTMs is quite faint. The oxidation of proteolytic enzymes can either induce or inhibit the signaling pathways that determine processes like cell fate and tissue remodeling. Further research into the exact redox-mediated PTMs in proteases will provide the tools for investigating the redox regulation of other proteases and signaling cascades. Alterations to proteolysis during oxidative stress can induce either protein aggregation or superfluous protein hydrolysis. Although proteostasis plays a crucial role in various neurodegenerative and age-related diseases, there is a lack of data linking these disorders to the redox regulation of proteolytic enzymes. Further investigations into oxidation-mediated PTMs and a definition of their roles in various inflammatory and age-related diseases could promote the development of novel therapeutics.
